# Synergistic effect of pulsed electric fields and temperature on the inactivation of microorganisms

**DOI:** 10.1186/s13568-021-01206-8

**Published:** 2021-03-23

**Authors:** Zeyao Yan, Li Yin, Chunjing Hao, Kefu Liu, Jian Qiu

**Affiliations:** Department of Light Sources and Illuminating Engineering; Academy for Engineering & Technology, Fudan University, Shanghai, 200433 China

**Keywords:** Pulsed electric fields, Initial temperature, Critical electric field intensity, Nucleic acid content, Protein content

## Abstract

Pulsed electric fields (PEF) as a new pasteurization technology played an important role in the process of inactivating microorganisms. At the same time, temperature could promote the process of electroporation, and achieve better inactivation effect. This article studied the inactivation effect of PEF on *Saccharomyces cerevisiae*, *Escherichia coli*, and *Bacillus velezensis* under different initial temperatures (room temperature-24 $\mathrm{^\circ{\rm C} }$, 30 $\mathrm{^\circ{\rm C} }$, 40 $\mathrm{^\circ{\rm C} }$, 50 $\mathrm{^\circ{\rm C} }$). From the inactivation results, it found temperature could reduce the critical electric field intensity for microbial inactivation. After the irreversible electroporation of microorganisms occurred, the nucleic acid content and protein content in the suspension increased with the inactivation rate because the cell membrane integrity was destroyed. We had proved that the electric field and temperature could promote molecular transport through the finite element simulation. Under the same initial temperature and electrical parameters (electric field intensity, pulse width, pulse number), the lethal effect on different microorganisms was *Saccharomyces cerevisiae* > *Escherichia coli* > *Bacillus velezensis.*

## Introduction

As we know, food safety and quality, which are humans pursuing health and quality of life concerns, are the dominating purpose of food testing and analysis (Weng and Neethirajan 2017). More and more consumers want the food they eat to be green, pollution-free, nutritious, as fresh as possible, and free from microbial contamination (Bisconsin-Junior et al. 2015). The traditional thermal pasteurization technology has a positive effect in controlling the quantity of microorganisms. However, thermal treatment damages the heat-sensitive substances and nutrients of food, motivating the development of new pasteurization technologies. The new pasteurization technology is expected to be energy-saving, efficient, and causes less damage to nutrients with minimal impact on food quality (Pasha et al. 2014, Wang et al. 2018, Yogesh, 2016). PEF processing is one of these non-thermal techniques that has been investigated during the last decades as an alternative for food pasteurization with less damage to food compared with the traditional thermal pasteurization technology. (Timmermans et al. 2014). The PEF pasteurization technology refers to applying a high pulsed voltage between two electrodes to form a uniform or non-uniform electric field region having a lethal effect on the microbes (Toepfl et al. 2007). When the liquid solution containing microbes flows through the intense electric fields region, the integrity of the cell membrane is damaged and the membrane permeability increases significantly (Huo et al. 2018). When microorganisms are exposed to PEF, electroporation occurs, the integrity of the membrane is destroyed, and genetic materials flow out (El Zakhem et al. 2007). Many studies have scientifically reported the effective application of PEF in food pasteurization and it promises to be a new method of pasteurization (Mahnic-Kalamiza et al. 2014).

In recent years, most reports have examined the effects of single electrical or environmental conditions on microbial inactivation (González-Arenzana et al. 2019, van Wyk et al. 2019). Factors affecting the results of microbial inactivation involve various aspects, including PEF parameters (pulse amplitude, duration, shape, number, rising edge, polarity), cell parameters (size, shape, orientation), and medium parameters (temperature, composition, conductivity, ionic strength, pH) (Bermúdez-Aguirre et al. 2012, Grahl and Markl 1996, Saulis, 2010). PEF has proven to be capable of inactivating microorganisms, and increasing the electric field intensity is the most effective way to increase the inactivation rate of microorganisms.

However, with constant load on the sample being processed, excessive electric voltage will generate excessive electric current, causing bubbles in the liquid food and abnormal breakdown. It is hoped to be combined with other methods that may synergistically inactivate microorganisms to achieve more sufficient pasteurization (Garner 2019, Vadlamani et al. 2018). Applying combined PEF-thermal treatments can induce membrane pore formation and damage, including lysis (El Zakhem et al. 2006). Choosing appropriate temperature and electrical parameters can effectively inactivate microorganisms, ensure food quality, extend the shelf life, and avoid breakdown of liquid food caused by excessive electric field intensity.

Some articles have reported the method of inactivating microorganisms that combine PEF with temperature in quasi-isothermal or non-isothermal conditions (Amiali et al. 2007, Saldaña et al. 2010). However, these reports do not provide details on the inactivation effect of pure temperature, the inactivation effect of PEF at room temperature, and the inactivation effect of combined temperature-PEF. Moreover, the microbial species treated are relatively single, and the research is not universal, so it is not possible to explain systematically the inactivation effect of combined thermal-PEF treatments.

In this study, we have investigated the inactivation of *Saccharomyces cerevisiae*, *Escherichia coli* and *Bacillus velezensis* under pure thermal treatment and pure PEF treatment (room temperature-24 $\mathrm{^\circ{\rm C} }$ as a standard temperature) and combined thermal-PEF treatments (30 $\mathrm{^\circ{\rm C} }$, 40 $\mathrm{^\circ{\rm C} }$, 50 $\mathrm{^\circ{\rm C} }$). These three microorganisms include fungi, bacteria, prokaryotes, eukaryotes, gram-positive bacteria, gram-negative bacteria, bacteria with spore structure and bacteria without spore structure, demonstrating the universality of combined temperature-PEF on microbial inactivation. At the same time, through the linear fitting, it can be obtained the temperature can reduce the critical electric field intensity, which can prove that temperature promotes the occurrence of irreversible electroporation. The content of nucleic acid and protein that spilled from the cells into the suspension is related to the inactivation rate of microorganisms.

## Materials and methods

### Cultivation of microorganisms

**Fig.1:**
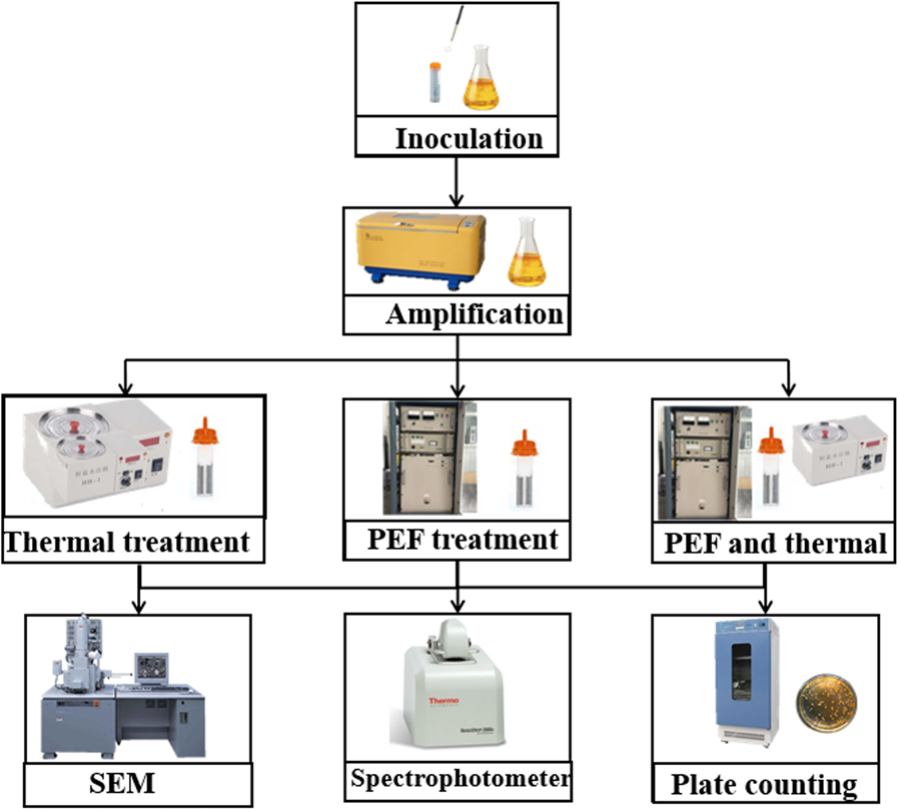
Experimental flow chart of the process including microbial culture, PEF and thermal treatments, plate counting, SEM detection, and spectrophotometer detection

*Saccharomyces cerevisiae* ATCC 201238 was inoculated into the prepared sterile yeast extract peptone dextrose medium (YPD) composed from 20 $\mathrm{g}/\mathrm{L}$ of peptone, 20 g/L of glucose, 10 g/L of yeast extract powder in a sterile environment, and cultured at 30 $\mathrm{^\circ{\rm C} }$ for approximately 12 h. The medium and culture conditions for culturing *Escherichia coli* CICC 10899 and *Bacillus velezensis* CICC 24434 were the same. The medium used was a nutrient broth medium composed of 5 $\mathrm{g}/\mathrm{L}$ sodium chloride, 10 $\mathrm{g}/\mathrm{L}$ peptone, and 3 $\mathrm{g}/\mathrm{L}$ beef extract powder, and the culture condition was 12 h at 37 $\mathrm{^\circ{\rm C} }$. We set the initial concentration of microorganisms at ${10}^{8}$ to ${10}^{9}$ CFU/ml. The electric conductivity and pH of the sample were measured by conductivity meter and pH meter, respectively, and they were 3.7mS/cm and 6.2. The specific flow chart of microbial culture and the system diagram of the whole experiment are shown in Fig. 1.

### Experiment system

**Fig.2:**
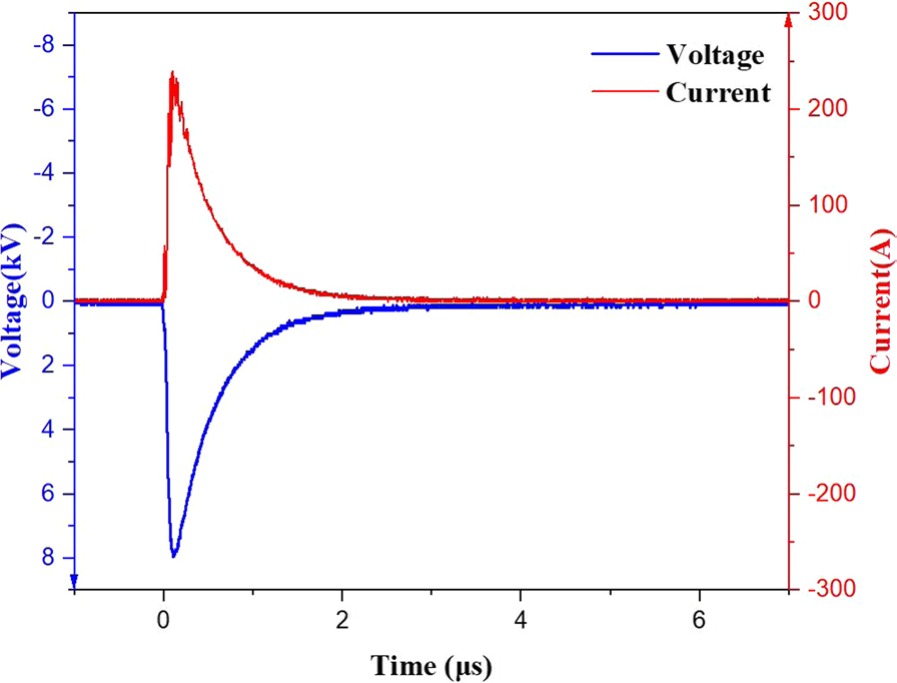
Typical voltage and current waveforms

The high voltage pulse generator was independently developed by Fudan University high power electronics research group (Shanghai, China). The high voltage pulse generator could output a high voltage ranging from 0 to 30 kV, the pulse width ranging from 0.5 to 2.5 $\mathrm{\mu s}$, and the frequency ranging from 0.1 to 10 Hz. Typical voltage and current waveforms used in this experiment are shown in Fig. 2. Since we chose exponential waves in our experiments, it was necessary to explain the definition of the pulse width. We defined the full width at half maximum of the pulse waveform as the pulse width, which was adjusted by changing the capacitance in the circuit.

In the experiment, the treatment chamber adopted a flat plate structure, the distance between the two plate electrodes was 4 mm, the width of a single plate was 10 mm, the height was 20 mm, the cross-sectional area was 200 mm^2^, and the total treatment volume was 0.8 mL. PEF pasteurization was suitable for high-resistance loads. Long electrode spacing and small facing areas were conducive to forming high-resistance loads. However, long electrode distances caused a reduction in the electric field intensity, which was negative for pasteurization. Considering all factors, the structural parameters of the above-mentioned treatment chamber were determined. The treatment chamber with the flat structure could form a uniform electric field, so the pasteurization effect was more uniform than other structures.

**Fig.3:**
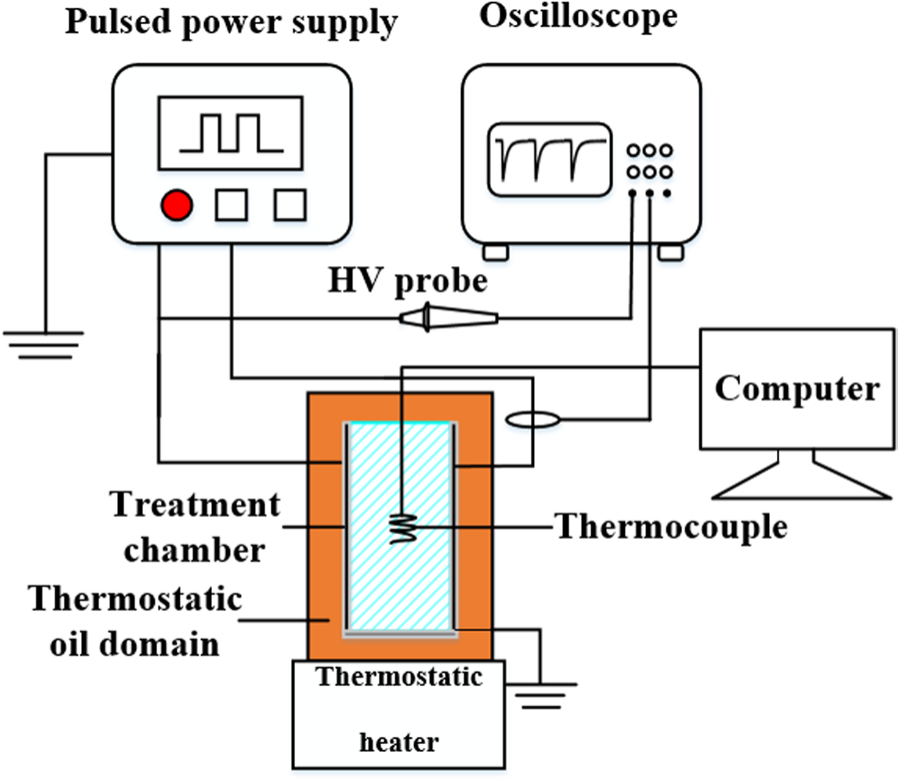
PEF and thermal treatment system

The experiment system is shown in Fig. 3. The voltage characteristics across the load and the current characteristics through the load were measured respectively by a four-channel oscilloscope (Agilent Technologies/DSO7104B) with an external high voltage probe (Tektronix/P6015A**/**1000:1) and a current loop (Pearson/4997**/** 100:1). We used a thermostatic heater to change the temperature of the thermostatic oil domain by adjusting the temperature of the thermostatic heater. The thermostatic oil field played two purposes in this system. One was to control the initial temperature of the sample to be treated by changing the temperature of the oil field. The other was to make the Joule heat generated during the action of the PEF dissipate as quickly as possible. A thermocouple (Shanghai Yanli Automation Instrument Corporation/ J-1**)** was used to detect the initial temperature of the sample and the final temperature after PEF treatment.

### Thermal treatment, PEF treatment, and PEF-thermal treatments

In order to study the influence of the temperature on the inactivation of microorganisms by PEF, we conducted three sets of experiments: (1) pure thermal inactivation, (2) pure PEF inactivation (at room temperature) and (3) PEF-thermal inactivation. We first changed the initial temperature of the sample, and then applied electric pulses to carry out the synergistic treatment of temperature and PEF, as shown in Table 1.

**Table 1:** The temperature rise under different initial temperatures and electric field intensities

Initial temperature ($\mathrm{^\circ{\rm C} }$)	Electric field intensity ($\mathrm{kV}/\mathrm{cm}$)	Temperature rise $\Delta \mathrm{T}$ ($\mathrm{^\circ{\rm C} }$)
Room temperature (24)	5	0.9
	10	4.7
	15	12.7
	20	20.3
	25	25.2
30	5	0.5
	10	4.0
	15	10.8
	20	17.2
	25	23.1
40	5	0.3
	10	1.4
	15	7.2
	20	14.0
	25	19.6
50	5	0.2
	10	1.1
	15	6.8
	20	12.6
	25	18.3

Pure thermal inactivation. High temperatures had a lethal effect on microorganisms because the high temperature could denature proteins structure of microorganisms, thereby killing microorganisms*.* In this paper, we chose a temperature range of 30 to $50\mathrm{^\circ{\rm C} }$ in increments of 5 $\mathrm{^\circ{\rm C} }$, because the temperature in this range was mild and would not have much impact on substances other than microorganisms. What needs to be emphasized was that the processing time for microbial inactivation by pure temperature was 1 min, which ensured the same time as the pure PEF treatment and PEF-thermal treatment.

Pure PEF inactivation. We investigated the effect of the three electrical parameters (electric field intensity, pulse width, pulse number) on microbial inactivation. The method used to study any of three electrical parameters was the controlled variable method. In this experiment, the electric field intensity adopted 5, 10, 15, 20, and 25 kV/cm. The pulse widths were 0.5, 1.2, 1.7, and 2.1 $\mathrm{\mu s}$. The number of pulses were 50, 100, 150, 200, 150, 300, 600, 900, and 1200. In all experiments, the frequency of PEF was chosen to be 10 Hz.

PEF-thermal inactivation. We first raised the samples to different initial temperatures (30 $\mathrm{^\circ{\rm C} }$, 40 $\mathrm{^\circ{\rm C} }$, 50 $\mathrm{^\circ{\rm C} }$) through the constant temperature oil domain, and then conducted PEF treatment at different initial temperatures. The selection of electrical parameters was consistent with that of pure PEF treatment. It took about 46 s for the untreated sample to grow from room temperature to the highest initial temperature 50 $\mathrm{^\circ{\rm C} }$. Therefore, before the PEF treatment, we waited for 1 min for the sample to reach the required initial temperature, and then performed PEF pasteurization. Similarly, the samples processed by pure thermal treatment and pure PEF treatment were placed in the treatment chamber for 1 min to reach the required temperature.

The effect of microbial inactivation under specific treatments was measured by the amount of viable microbial by the plate counting before and after treatments. The plate counting was the most suitable way to define the effect of inactivation because it prevented pore healing, which could lead to suspended animation of microorganisms (Rols and Teissie 1990). Defined survival rate $S=Log(N/{N}_{0})$ as a criterion for judging inactivation effect, *N* (CFU/mL) is the number of viable microbial after processing, and *N*_*0*_ (CFU/mL) is the number of viable microbial before processing. Each experiment was repeated at least three times, and the mean value of the three experimental results were calculated to determine the final experimental results, including error bars being by standard error. Statistical analysis was performed using one-way analysis of variance (ANOVA; P < 0.05) followed by Tukey’s post hoc tests.

### Determination of nucleic acid and protein content in suspension

Microorganisms underwent electroporation under the action of PEF. The integrity of the cell membrane was destroyed, and the nucleic acids and proteins inside the cell spilled into the suspension. The bases that made up the nucleic acid molecule had the characteristic of ultraviolet absorption due to the aromatic ring structure. The absorption value was between 250 and 270 nm and the maximum absorption wavelength was 260 nm (Huo et al. 2017). Therefore, the content of nucleic acid could be calculated by measuring the light absorption value of nucleic acid at 260 nm. The protein contained conjugated double bonds tyrosine and tryptophan, and the absorbance at 280 nm was used to estimate the protein content. The content of nucleic acid and protein in the suspension were detected by spectrophotometer (One Drop/OD 1000). First, the samples were centrifuged at 5000 rad/min for 15 min. Then, 2 $\mathrm{\mu l}$ of supernatant were placed in the spectrophotometer to detect the content of nucleic acid and protein by absorbance of different wavelengths. Each sample was tested three times to determine the final nucleic acid and protein concentration.

### Data fitting analysis

Based on the least squares fit method, the data were fitted and analyzed by OirginPro software (Version 2017). ${R}^{2}$ was the statistical parameter to show the goodness of the fits.

## Results

### Electric field distribution in the treatment chamber

**Fig.4:**
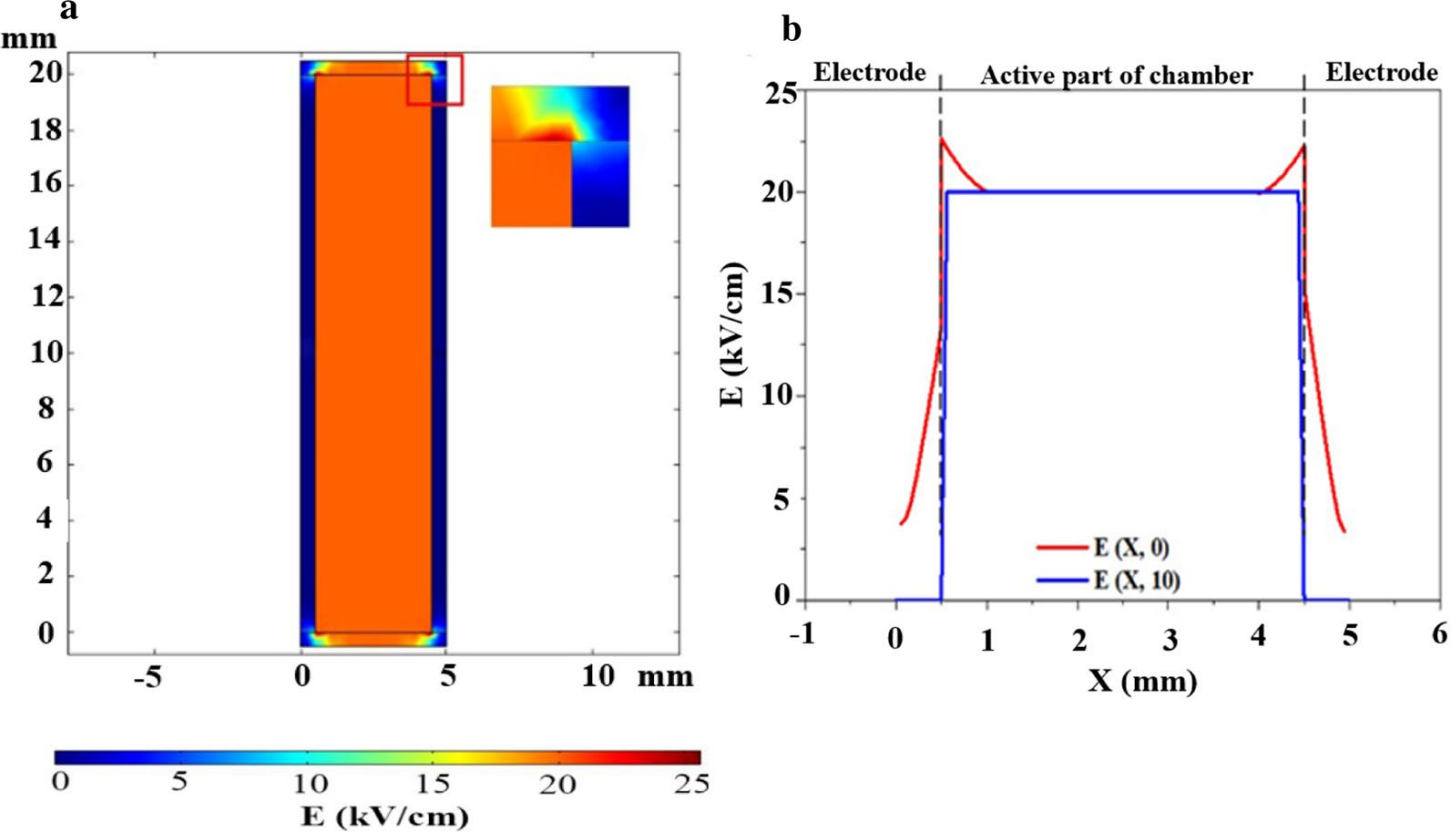
The distribution of the electric field (**a** in XY cross section plane, **b** along X axis.)

Different treatment chambers would produce different load characteristics, resulting in different electric field distributions, which could have different effects on inactivation effects. The uniform electric field generated by the parallel plate electrodes could avoid the problem of under-treatment or over-treatment, which was crucial for food pasteurization compared with the non-uniform electric field generated by coaxial electrodes (Huang and Wang 2009). The electric field distribution in the treatment chamber was obtained through the numerical simulation in Fig. 4a. Simulations were performed using finite element simulation software COMSOL Multiphysics v5.3a. The boundary conditions of this model was electric insulation, the relative dielectric constant of the material between the parallel plates was 80, the conductivity was 3.7 $\mathrm{mS}/\mathrm{cm}$, and the applied voltage was 8 $\mathrm{kV}$. The simulated electric field was uniformly distributed throughout the chamber except for distortion along the corners and edges where the electrodes were in contact with the insulated medium to produce a strong electric field intensity at the tip. The distorted electric field region occupied a quite small part of the whole electric field region, and its influence could be ignored. The distorted electric field region was enlarged and could be seen in the upper right corner of Fig. 4a. From Fig. 4b, the homogeneous electric field (E = 20 $\mathrm{kV}/\mathrm{cm}$) was obtained within the active part of the chamber between the two electrodes, while the electric field outside the active region is zero (the blue line). There were obvious electric field distortions at the corner of the chamber, resulting in a significant increase in the electric field (the red line).

### Analysis of microbial inactivation

**Fig.5:**
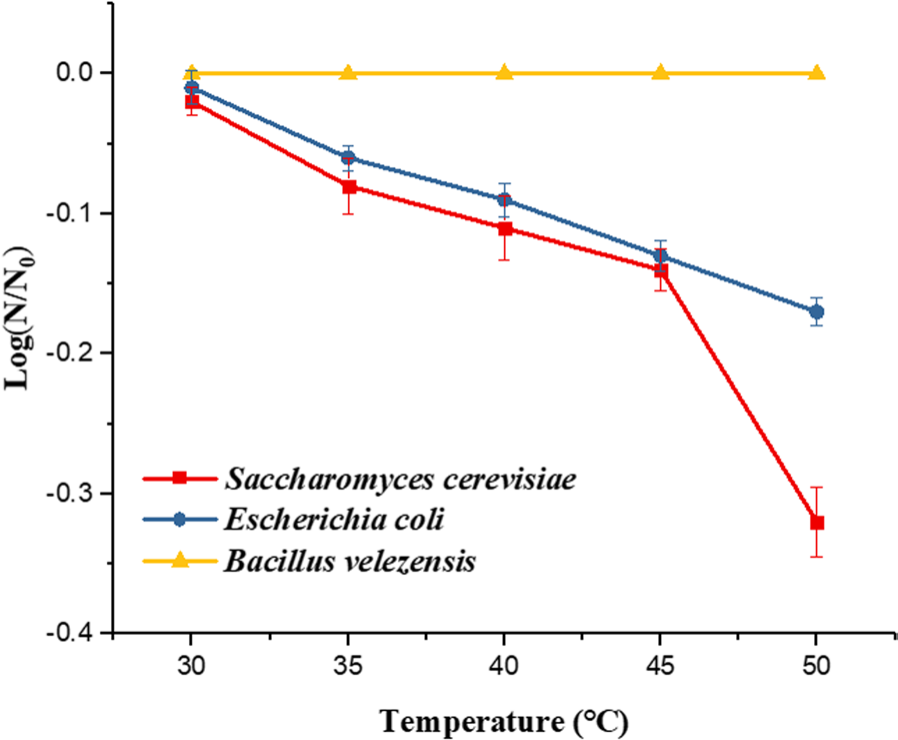
The survival rate of *Saccharomyces cerevisiae, Escherichia coli,* and *Bacillus velezensis* changes with temperature

As shown in Fig. 5, we got the curve of the survival rate of three microorganisms (*Saccharomyces cerevisiae, Escherichia coli*, and *Bacillus velezensis*) with temperature. The survival rate of *Saccharomyces cerevisiae* after pure thermal treatment decreased by 0.02, 0.11 and 0.32 logs at the processing temperature of 30, 40 and 50 $\mathrm{^\circ{\rm C} }$. The survival rate of *Escherichia Coli* decreased by 0.01, 0.09, and 0.17 logs at the processing temperature of 30, 40, and 50 $\mathrm{^\circ{\rm C} }$. Because of *Bacillus velezensis* with spore structure, which made it heat-resistant, there was no change in the survival rate of *Bacillus velezensis* within this temperature range.

**Fig.6:**
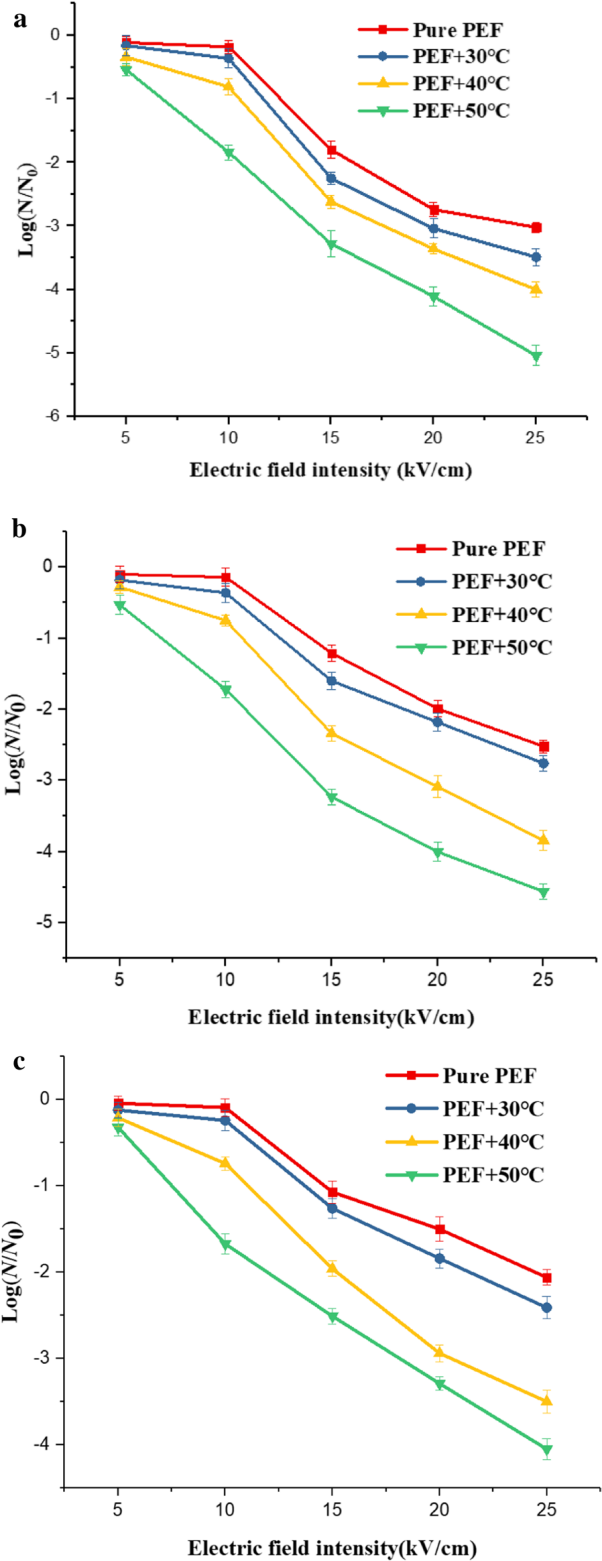
The effect of electric field intensity on the survival rate at different temperatures (**a** *Saccharomyces cerevisiae,* **b** *Escherichia coli*, **c** *Bacillus velezensis*)

The effect of electric field intensity on microbial survival rate. The experimental conditions to study the effect of electric field intensity on microbial inactivation as follows: the electric field intensity varied from 5 to 25 $\mathrm{kV}/\mathrm{cm}$, increasing at intervals of 5 $\mathrm{kV}/\mathrm{cm}$, under the condition of pulse width of 0.5 $\mathrm{\mu s}$, pulse number of 600 and the pulse frequency of 10 $\mathrm{Hz}$. In Fig. 6a, the curve drawn in red square described the change of *Saccharomyces cerevisiae* survival rate with the electric field intensity. The electric filed intensity changed from 5 to 25 $\mathrm{kV}/\mathrm{cm}$, and the survival rate correspondingly changed from − 0.11 to − 3.02 causing approximately 2.9 logs change. The survival rate decreased statistically significantly with the increase of electric field intensity (P < 0.05). In Fig. 6b, we could find that the survival rate of *Escherichia Coli* decreased statistically significantly with the increase of the electric field intensity from the red square curve (P < 0.05). At 25 $\mathrm{kV}/\mathrm{cm}$, the survival rate of *Escherichia Coli* was − 2.52, which was more than that of *Saccharomyces cerevisiae.* The red square curve in Fig. 6c described the influence of electric field intensity on the survival rate of *Bacillus velezensis*. The survival rate of *Bacillus velezensis* decreased statistically significantly with the increase of the electric field intensity (P < 0.05). Compared with *Saccharomyces cerevisiae* and *Escherichia coli*, the PEF had the worst inactivation effect on *Bacillus velezensis*, and the survival rate corresponding to the electric field intensity of 25 $\mathrm{kV}/\mathrm{cm}$ was − 2.06.

**Fig.7:**
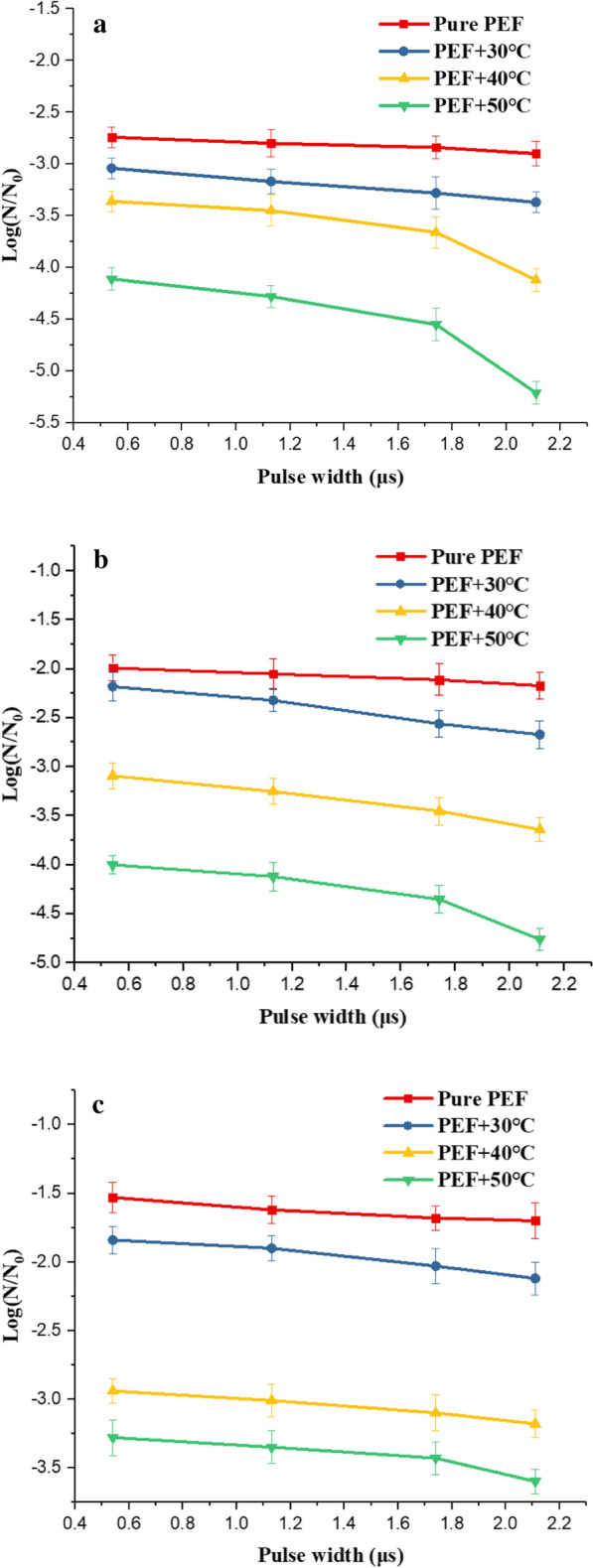
The effect of pulse width on the survival rate at different temperatures (**a** *Saccharomyces cerevisiae,* **b Escherichia coli,** **c** *Bacillus velezensis*)

The effect of pulse width on microbial survival rate. Under the condition of electric field intensity of 20 $\mathrm{kV}/\mathrm{cm}$, pulse number of 600, pulse frequency of 10 Hz, we studied the microbial survival rate as a function of pulse width from 0.5 to 2.1 $\mathrm{\mu s}$. From Fig. 7, the red square curve described that the survival rates of *Saccharomyces cerevisiae*, *Escherichia coli* and *Bacillus velezensis* decreased with the increase of the pulse width (P > 0.05). The pulse width changed from 0.5 to 2.1 $\mathrm{\mu s}$, the survival rate of *Saccharomyces cerevisiae* was changed from − 2.74 to − 2.90, the survival rate of *Escherichia coli* was changed from − 1.99 to − 2.17, and the survival rate of *Bacillus velezensis* changed from − 1.53 to − 1.70. The increase in pulse width could reduce the survival rate of microorganisms, but the degree of change in survival rate is not significant statistically (P > 0.05). In the equivalent circuit model of a single cell, the cell membrane was equivalent to a capacitor. The charging time constant of the cell membrane was determined by formula ${\tau }_{m}=r{C}_{m}(\frac{1}{{2\sigma }_{e}}+\frac{1}{{\sigma }_{i}})$ (Schoenbach, 2010). Where ${\tau }_{m}$ is the charging time constant of the cell membrane, $r$ is the cell radius, ${C}_{m}$ is the membrane capacitance of the cell membrane, ${\sigma }_{e}$ is the conductivity of external medium, ${\sigma }_{i}$ is cytoplasmic conductivity. The cell membrane charging time constant was estimated to be 1 $\mathrm{\mu s}$, which was very close to the order of the pulse width we used. Changing the pulse width around the charging time constant had minimal effect on the survival rate of microorganisms (Kotnik and Pucihar 2010).

**Fig.8:**
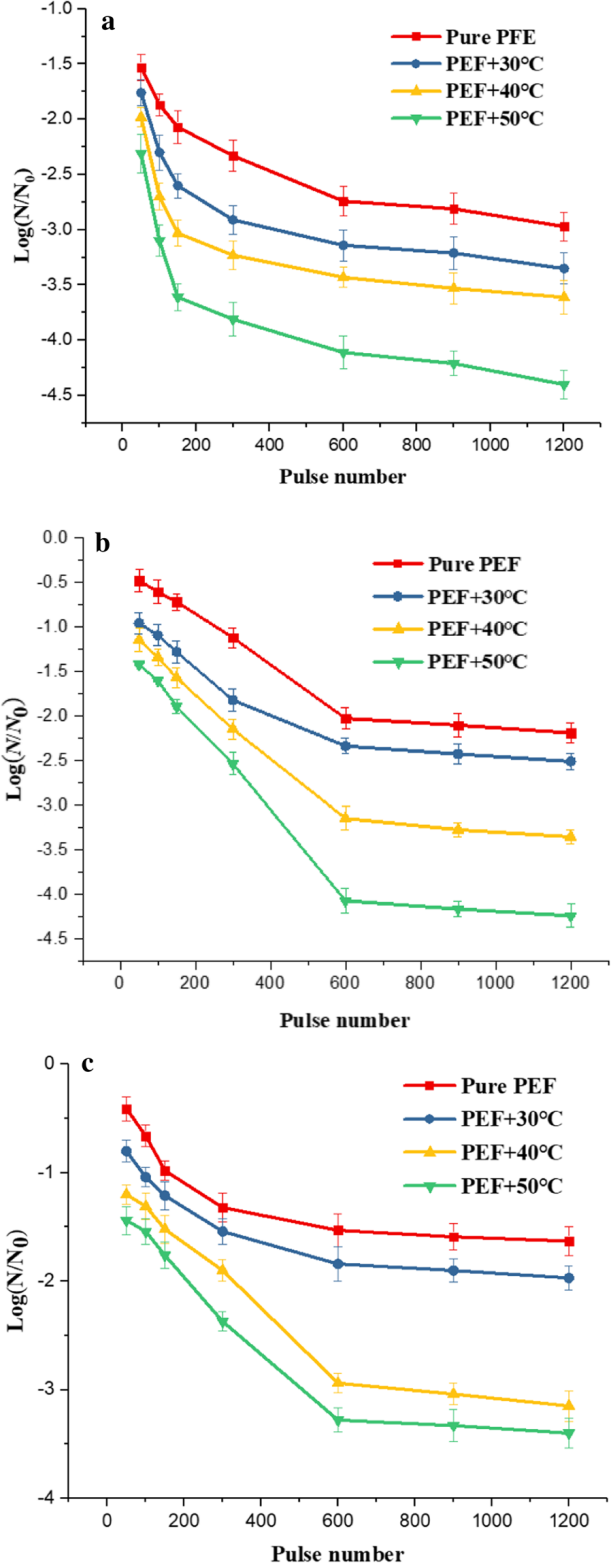
The effect of pulse number on the survival rate at different temperatures (**a** *Saccharomyces cerevisiae,* **b*** Escherichia coli*, **c*** Bacillus velezensis*)

The effect of pulse number on microbial survival rate. Consistent with the method of studying electric field intensity and pulse width, we investigated the effect of pulse number on microbial inactivation. The experimental electrical parameters were as follows, electric field intensity of $20\mathrm{ kV}/\mathrm{cm}$, pulse width of 0.5 $\mathrm{\mu s}$, and pulse frequency of 10 Hz. We could find that increasing pulse number could effectively reduce the survival rate of *Saccharomyces cerevisiae*, *Escherichia coli* and *Bacillus velezensis* from the red square curve in Fig. 8*.* After pulse number increased to 600, the survival rate still reduced, but in a much slower rate. The survival rate did not change significant statistically (P > 0.05). Therefore, when we studied the effect of electric field intensity and pulse width on the survival rate, we chose to set pulse number to 600.

The effect of initial temperature and electric field intensity on microbial survival rate. In Fig. 6.a, the increase in temperature and electric field intensity caused survival rate of *Saccharomyces cerevisiae* to decrease. At 25 $\mathrm{kV}/\mathrm{cm}$ and 50 $\mathrm{^\circ{\rm C} }$, the survival rate decreased by 5.04 logs, which was much greater than 0.32 logs obtained by pure thermal treatment at 50 $\mathrm{^\circ{\rm C} }$ and 3.02 logs obtained by pure PEF treatment at 25 $\mathrm{kV}/\mathrm{cm}$.

At 25 $\mathrm{kV}/\mathrm{cm}$, by increasing the initial temperature of the sample, the survival rate of *Saccharomyces cerevisiae* could be effectively reduced, and the survival rate changed from − 3.02( room temperature-24 $\mathrm{^\circ{\rm C} }$) to − 5.04 (50 $\mathrm{^\circ{\rm C} }$). In Fig. 6b, c, we could find that the inactivation effect of temperature and electric field intensity on *Escherichia coli* and *Bacillus velezensis* had the same trend as *Saccharomyces cerevisiae.* When *Escherichia coli* and *Bacillus velezensis* were exposed to higher electric field intensity and temperature, the corresponding survival rate became lower. At 25 $\mathrm{kV}/\mathrm{cm}$ and 50 $\mathrm{^\circ{\rm C} }$, the survival rate of *Escherichia coli* decreased by 4.56 logs, which was much greater than 0.17 logs treated by pure thermal treatment (50 $\mathrm{^\circ{\rm C} }$) and 2.52 logs obtained by pure PEF treatment at 25 $\mathrm{kV}/\mathrm{cm}$. At 25 $\mathrm{kV}/\mathrm{cm}$ and 50 $\mathrm{^\circ{\rm C} }$, the survival rate of *Bacillus velezensis* decreased by 4.05 logs. Inversely, the survival rate of *Bacillus velezensis* decreased by 2.06 logs obtained by pure PEF treatment at 25 $\mathrm{kV}/\mathrm{cm}$. It was worth mentioning that pure thermal treatment did not have the inactivation effect on *Bacillus velezensis*, but a higher initial temperature promoted the inactivation effect on *Bacillus velezensis* by the PEF.

The effect of initial temperature and pulse width on microbial survival rate. In Fig. 7a, the survival rate of *Saccharomyces cerevisiae* changed significantly from − 4.11 to − 5.21 when the pulse width changed from 0.5 to 2.1 $\mathrm{\mu s}$ at 50 $\mathrm{^\circ{\rm C} }$ (P < 0.05). This change in survival rate was obvious compared with that in pure PEF inactivation. The same pulse width change only caused the survival rate to change from − 2.74 to − 2.9 under pure PEF treatment, which was not significant statistically (P > 0.05). From Fig. 7b, c, we found that increasing the pulse width and initial temperature could decrease the survival rate of *Escherichia coli*, and *Bacillus velezensis*. At 50 $\mathrm{^\circ{\rm C} }$, the pulse width changed from 0.5 to 2.1 $\mathrm{\mu s}$, the survival rate of *Escherichia coli* was changed from − 4.01 to − 4.76, and the survival rate of *Bacillus velezensis* changed from − 3.28 to − 3.61.

The effect of initial temperature and pulse number on microbial survival rate. Figure 8a–c respectively described the survival rate curve of *Saccharomyces cerevisiae*, *Escherichia coli*, and *Bacillus velezensis* under different initial temperature and pulse number. The initial temperature and pulse number reduced the survival rate of microorganisms, which was consistent with the influence of electric field intensity and pulse width on microbial inactivation. When the pulse number was less than 600, the microbial survival rate decreased statistically significantly with the increase of pulse number (P < 0.05). After the pulse number reached 600, the decreasing trend of the microbial survival rate became slower with the increase of the pulse number.

We studied the inactivation of *Saccharomyces cerevisiae*, *Escherichia coli*, and *Bacillus velezensis* by three different electrical parameters (electric field intensity, pulse width, pulse number) under different initial temperatures. Under the same initial temperature and electrical parameters, *Saccharomyces cerevisiae* had the lowest survival rate, followed by *Escherichia coli*, and *Bacillus velezensis* had the highest survival rate. This was because *Saccharomyces cerevisiae* was the largest and had the strongest sensitivity to electric fields (DeBruin and Krassowska 1999, Krassowska and Filev 2007). *Escherichia coli* was a gram-negative bacterium, and it was moderately sensitive to electric fields (Evrendilek et al. 2000). *Bacillus velezensis* had the least sensitive to electric fields because it was a gram-positive bacterium (Heinz et al. 2001, Heinz et al. 2009). What is more, *Bacillus velezensis* had spore structure, which was resistant to electric field.

### The synergistic effect of PEF-thermal treatment

PEF-thermal treatments induced greater inactivation than either PEF or thermal treatments individually. Here we discussed a more important issue. We compared the inactivation effect of combined PEF-thermal treatments with the sum of the inactivation effect of their two separate treatments. Based on Eq. (1), the sum survival rate ${S}_{T+PEF}$ of pure temperature and pure PEF treatments could be obtained.

1 $$S_{T + PEF} = {\text{Log}}\left( {\left( {\frac{N}{{N_{0} }}} \right)_{T} \times \left( {\frac{N}{{N_{0} }}} \right)_{PEF} } \right) = {\text{Log}}\left( {N/N_{0} } \right)_{T} + {\text{Log}}\left( {{\text{N}}/N_{0} } \right)_{PEF} = S_{T} + S_{PEF}$$

where ${S}_{T}$ is the survival rate under pure thermal treatments, and ${S}_{PEF}$ is the survival rate under pure PEF. All the results were obtained under the experimental conditions of pulse width of 0.5 $\mathrm{\mu s}$, pulse number of 600 and frequency of 10 Hz. We defined the synergy coefficient *k* to characterize the synergistic effect of PEF-thermal treatments on microorganism inactivation.

2 $$k = \frac{S}{{S_{T + PEF} }} = \frac{{Log\left( {N/N_{0} } \right)}}{{Log\left( {N/N_{0} } \right)_{T} + Log\left( {N/N_{0} } \right)_{PEF} }}$$

$S$ is the survival rate of combined PEF-thermal treatments.

**Fig.9:**
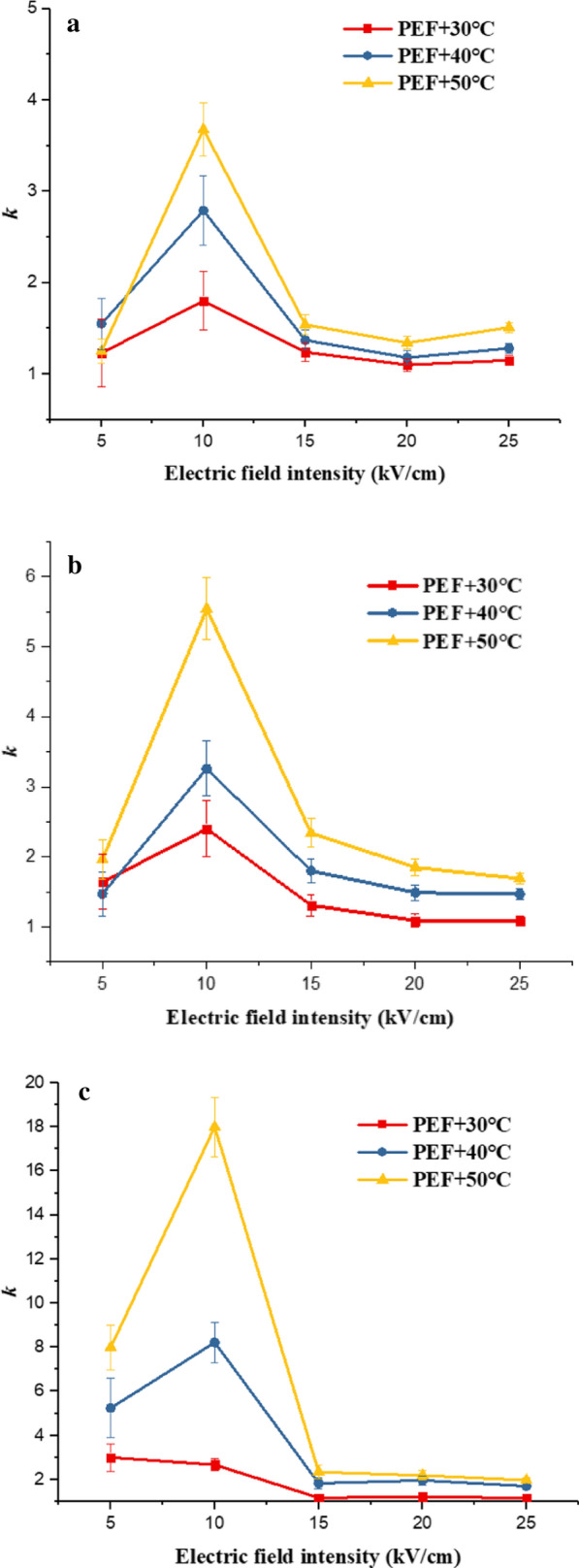
Synergy coefficient under different electric field intensities and initial temperatures (**a** *Saccharomyces cerevisiae,* **b*** Escherichia coli*, **c*** Bacillus velezensis*)

As shown in Fig. 9, we could find that *k* was bigger than 1, which approved the synergistic effect between temperature and PEF. The inactivation effect of combined thermal-PEF treatment was better than the sum inactivation effect of pure thermal treatment and pure PEF treatment. For *Saccharomyces cerevisiae*, *Escherichia coli*, and *Bacillus velezensis*, the electric parameter and initial temperature of the maximum synergy coefficient were 10 $\mathrm{kV}/\mathrm{cm}$ and 50 $\mathrm{^\circ{\rm C} }$. Especially for *Bacillus velezensis*, pure thermal treatment of 50 $\mathrm{^\circ{\rm C} }$ had no lethal effect on *Bacillus velezensis*. However, we control the initial temperature to 50 $\mathrm{^\circ{\rm C} }$, combined with PEF treatment, and the survival rate was statistically significantly decreased compared with pure PEF treatment (P < 0.05).

### Temperature rise caused by Joule heating

When the PEF was used to inactivate microorganisms, the current passing through the suspension generated Joule heating, thereby increasing the temperature of the suspension. The temperature change of the suspension had a very important relationship with the quality of the liquid food, so it was necessary to detect the temperature of the sample after the PEF treatment. Here we studied the changes in temperature after pure PEF treatment and combined PEF-thermal treatment when the electric field intensity was a variable. The temperature rise under different conditions is shown in Table 1.

### Detection of nucleic acid content and protein content in suspension

**Fig.10:**
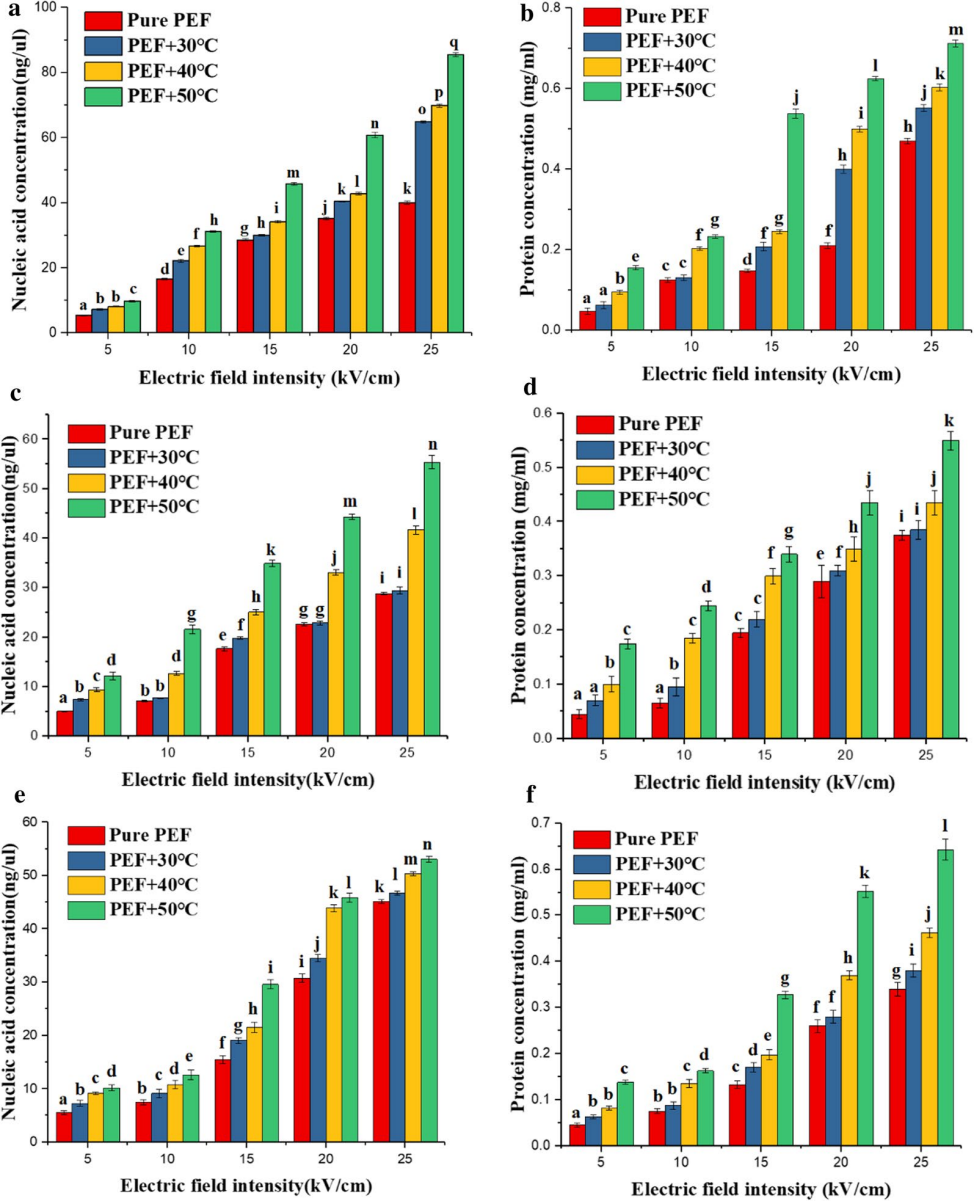
Nucleic acid content and protein content in suspension under different electric field intensities and initial temperatures (**a** *Saccharomyces cerevisiae*, nucleic acid, **b** *Saccharomyces cerevisiae*, protein, **c** *Escherichia coli*, nucleic acid, **d** *Escherichia coli*, protein, **e** *Bacillus velezensis*, nucleic acid, and **f** *Bacillus velezensis*, protein). Different letters (a-q) on the column indicate that values are significantly different (p < 0.05)

As shown in Fig. 10, we used a spectrophotometer to measure the content of nucleic acid and protein in the suspension under different initial temperatures and electric field intensities. The content of nucleic acid and protein in the suspension increased statistically significantly with the increase of the electric field intensity and the initial temperature (P < 0.05). The lower the survival rate of microorganisms, the higher the nucleic acid and protein content in the suspension. The nucleic acid content and protein content in the suspension were negatively correlated with the survival rate of microorganisms. After microorganisms were treated by the PEF, the permeability of the cell membrane increased, and nucleic acids and proteins overflowed to the outside of the cell, destroying the homeostasis of the cell and the normal physiological process of the cell, and resulting in inactivation of microorganisms.

### The effect of temperature and electric field on molecular transport

The material inside and outside the cell was exchanged through the cell membrane. When electroporation occurred, the integrity of the cell membrane was destroyed, and genetic material and proteins flowed out of the cell. Molecular transport in porous media under an electric field maintained the Nernst-Planck equation (Li and Lin 2011).

3 $$\frac{\partial c}{{\partial t}} = \nabla \cdot \left( {D\nabla c + \frac{DzF}{{RT}}cE} \right)$$

4 $$D = \mu_{P} k_{B} T$$

where *c* is the concentration of molecules, $D$ is the diffusion coefficient, ${\mu }_{P}$ is molecules velocity, ${k}_{B}$ is Boltzmann constant, z is the charge number, $F$ is Faraday’s constant, $R$ is the ideal gas constant, $T$ is absolute temperature and $E$ is electric field intensity.

In our model, the electroporated cell that had an external diameter of 5 $\mathrm{\mu m}$, 5 nm thickness cell membrane with a pore of 10 nm radius. We assumed that the particles were going long-lived pores. The boundary condition was electric insulation and the parameter settings are shown in Table 2 (Gómez et al. 2014, Jayasooriya and Nawarathna 2017, Roth et al. 2014). We had carried out simulation research of transport of molecules pass through pores in the cell membrane on the conditions of (1). 30 $\mathrm{^\circ{\rm C} }$, 5 $\mathrm{kV}/\mathrm{cm}$, (2). 40 $\mathrm{^\circ{\rm C} }$, 5 $\mathrm{kV}/\mathrm{cm}$, (3). 50 $\mathrm{^\circ{\rm C} }$, 5 $\mathrm{kV}/\mathrm{cm}$, (4). 30 $\mathrm{^\circ{\rm C} }$, 15 $\mathrm{kV}/\mathrm{cm}$, (5). 40 $\mathrm{^\circ{\rm C} }$, 15 $\mathrm{kV}/\mathrm{cm}$, (6). 50 $\mathrm{^\circ{\rm C} }$, 15 $\mathrm{kV}/\mathrm{cm}$, (7). 30 $\mathrm{^\circ{\rm C} }$, 25 $\mathrm{kV}/\mathrm{cm}$, (8). 40 $\mathrm{^\circ{\rm C} }$, 25 $\mathrm{kV}/\mathrm{cm}$, and (9). 50 $\mathrm{^\circ{\rm C} }$, 25 $\mathrm{kV}/\mathrm{cm}$.

**Table 2:** Model parameters

symbol/unit	Value	Definition
${c}_{1}/mol\bullet {m}^{-3}$	$2\times {10}^{-4}$	Initial concentration of molecules inside cell
${c}_{2}/mol\bullet {m}^{-3}$	0	Initial concentration of molecules outside cell
${\mu }_{P1}/m\bullet {s}^{-1}\bullet {N}^{-1}$	$2.04\times {10}^{-17}$	Molecules velocity inside cell
${\mu }_{P2}/m\bullet {s}^{-1}\bullet {N}^{-1}$	$2.04\times {10}^{-15}$	Molecules velocity in a pore
${\mu }_{P3}/m\bullet {s}^{-1}\bullet {N}^{-1}$	$2.04\times {10}^{-15}$	Molecules velocity outside cell
${k}_{B}/J\bullet {K}^{-1}$	${1.38\times 10}^{-23}$	Boltzmann constant
$\mathrm{z}/1$	− 1	The charge number
$F{/C\bullet mol}^{-1}$	96,485	Faraday’s constant
$R/J\bullet {mol}^{-1}{\mathrm{K}}^{-1}$	8.3145	Gas constant

**Fig.11:**
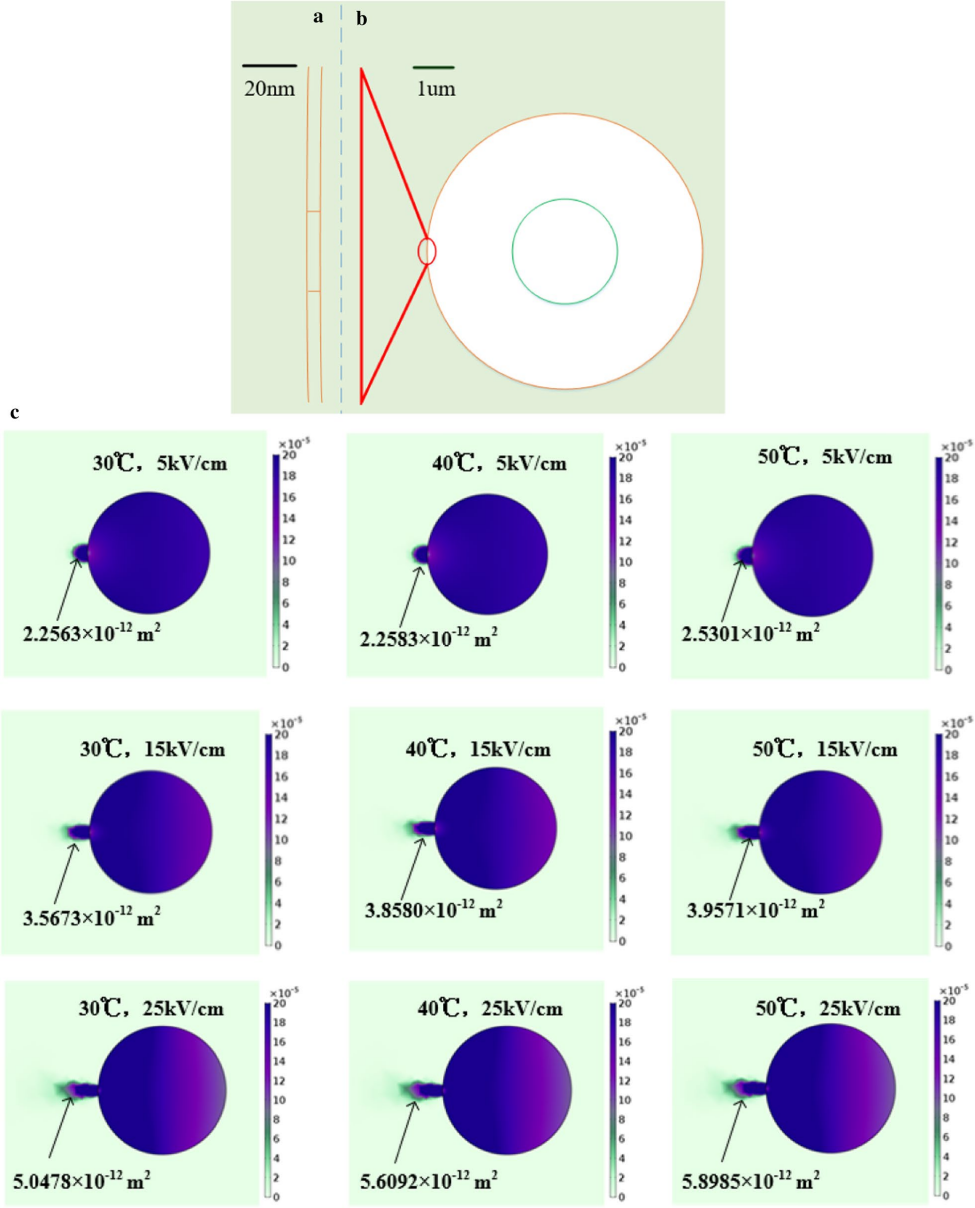
The effect of temperature and electric field on molecular transport. (**a** Zoomed view of the pore. **b** Cell. **c** The spatial distribution of molecular concentration ($\mathrm{mol}/{\mathrm{m}}^{3}$))

In Fig. 11, we presented the different the spatial distribution of molecular concentration ($\mathrm{mol}/{\mathrm{m}}^{3}$) under nine different conditions at 2 s. We calculated the area where the concentration of the extracellular molecule was greater than $2\times {10}^{-5}\mathrm{ mol}/{\mathrm{m}}^{3}$. We could find that increasing the electric field intensity and temperature promoted the transport of intracellular materials, and the concentration of extracellular molecules was higher. Both temperature and electric field accelerated the transport of molecules, destroyed the integrity of cells, increased the degree of irreversible electroporation, and increased the inactivation rate of microorganisms. According to Eq. (3), we could find that the diffusion of molecules was proportional to temperature and electric field intensity, which was consistent with simulation results. At the same time, the simulation results further proved that in our previous part of the experiment, increasing the temperature and electric field intensity could increase the nucleic acid content and protein content in the suspension.

### Microbial inactivation kinetics of combined PEF-thermal treatment

The initial model of survival rate as a function of electric field intensity was proposed by Huelsheger et al. (1981). The survival rate *S* was related to the electric field intensity by

5 $$S = - b_{e} \left( {E - E_{C} } \right)$$

**Fig.12:**
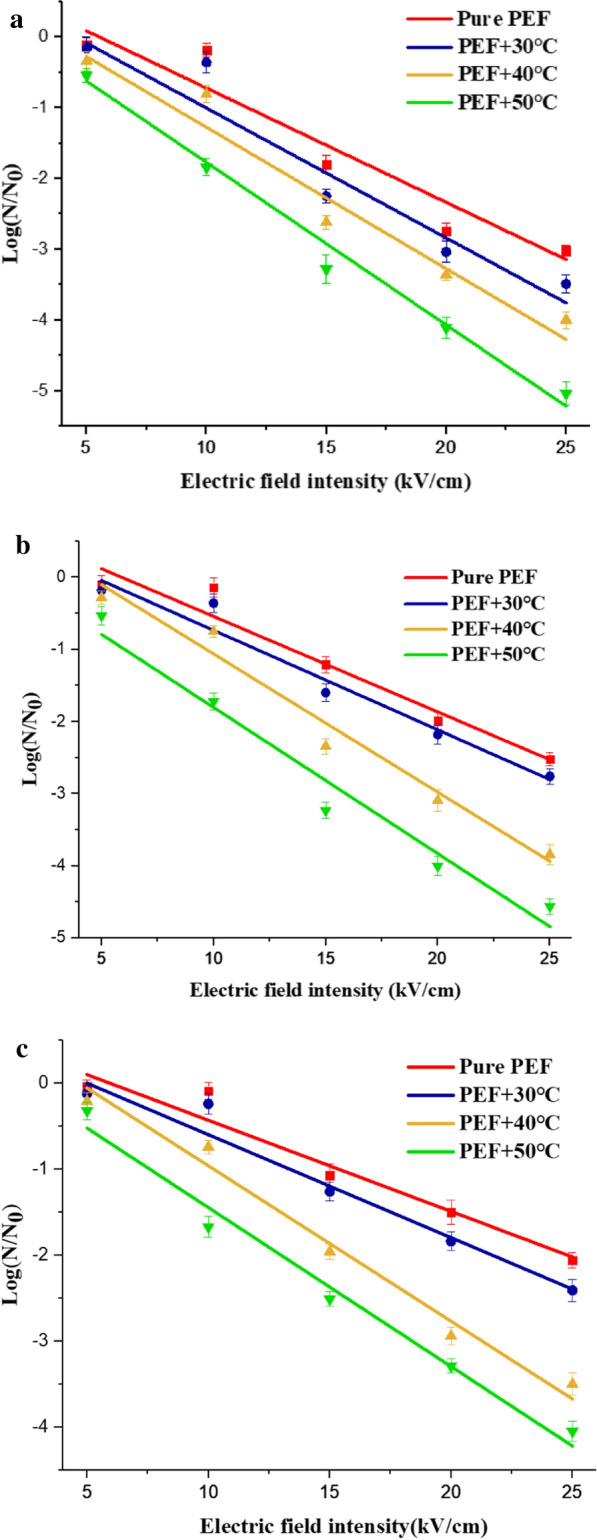
Critical electric field intensity fitting analysis at different initial temperatures (**a** *Saccharomyces cerevisiae,* **b*** Escherichia coli*, **c*** Bacillus velezensis*)

Where ${b}_{e}$ is a regression coefficient and ${E}_{c}$ is the electric field intensity at the extrapolated survival fraction of 100$\mathrm{\%}$. We fitted the experiment data of microbial inactivation under different initial temperatures and electric field intensities according to Eq. (5), and the results obtained by fitting were shown in Fig. 12. The value of parameters ${b}_{e}$ and ${E}_{c}$ are shown in Table 3. The increasing temperature caused ${E}_{c}$ to decrease and ${b}_{e}$ to increase. For *Saccharomyces cerevisiae*, ${E}_{c}$ decreased from 5.632 $\mathrm{kV}/\mathrm{cm}$ to 1.859 $\mathrm{kV}/\mathrm{cm}$ and ${b}_{e}$ increased from 0.1676 $\%\bullet \mathrm{cm}/\mathrm{kV}$ to 0.2254$\%\bullet \mathrm{cm}/\mathrm{kV}$. The increasing temperature reduced the critical electric field intensity of *Saccharomyces cerevisiae* inactivation and increased the slope of *Saccharomyces cerevisiae* inactivation, which represented a better and faster inactivation effect. Similarly, for *Escherichia coli* and *Bacillus velezensis*, the higher initial temperature led to a decrease in critical electric field intensity and an increase in slope of microbial inactivation. ${E}_{c}$ for *Escherichia coli* decreased from 6.091 $\mathrm{kV}/\mathrm{cm}$ to 1.422$\mathrm{kV}/\mathrm{cm}$, and ${b}_{e}$ increased from 0.1338 $\%\bullet \mathrm{cm}/\mathrm{kV}$ to 0.2068$\%\bullet \mathrm{cm}/\mathrm{kV}$. ${E}_{c}$ for *Bacillus velezensis* decreased from 6.266 $\mathrm{kV}/\mathrm{cm}$ to 1.960 $\mathrm{kV}/\mathrm{cm}$, and ${b}_{e}$ increased from 0.1090 $\%\bullet \mathrm{cm}/\mathrm{kV}$ to 0.1816$\%\bullet \mathrm{cm}/\mathrm{kV}$. The higher temperature reduced the value of the critical electric field intensity, irreversible electroporation was more likely to occur, and the corresponding survival rate was lower. Under the same initial temperature and electrical parameters, we could get that the critical electric field intensity of *Bacillus velezensis* was the largest, and that of *Saccharomyces cerevisiae* was the smallest. This also proved that PEF had the strongest inactivation effect on *Saccharomyces cerevisiae* and the weakest effect on *Bacillus velezensis.*

**Table 3:** Parameters from regression analysis for experiments

Experiment conditions	${b}_{e}(\%\bullet \mathrm{cm}/\mathrm{kV})$		${E}_{c}(\mathrm{kV}/\mathrm{cm})$		${R}^{2}$
a. *Saccharomyces cerevisiae*					
Pure PEF	0.1676		5.632 $\pm$ 2.537		0.9255
PEF + 30 $\mathrm{^\circ{\rm C} }$	0.1868		5.043 $\pm$ 2.911		0.9339
PEF + 40 $\mathrm{^\circ{\rm C} }$	0.1974		3.732 $\pm$ 2.171		0.9597
PEF + 50 $\mathrm{^\circ{\rm C} }$	0.2254		1.859 $\pm 0.698$		0.9875
b*. Escherichia coli*					
Pure PEF	0.1338	6.091 $\pm$ 1.998		0.9523	
PEF + 30 $\mathrm{^\circ{\rm C} }$	0.1396	4.857 $\pm$ 1.844		0.9616	
PEF + 40 $\mathrm{^\circ{\rm C} }$	0.1892	4.112 $\pm$ 1.614		0.9736	
PEF + 50 $\mathrm{^\circ{\rm C} }$	0.2068	1.422 $\pm$ 1.914		0.9683	
c*. Bacillus velezensis*					
Pure PEF	0.1090	6.266 $\pm 1.941$		0.9530	
PEF + 30 $\mathrm{^\circ{\rm C} }$	0.1236	5.502 $\pm$ 1.699		0.9640	
PEF + 40 $\mathrm{^\circ{\rm C} }$	0.1756	4.351 $\pm$ 1.575		0.9831	
PEF + 50 $\mathrm{^\circ{\rm C} }$	0.1816	1.960 $\pm$ 1.193		0.9837	

### Scanning electron microscope (SEM) characteristics

**Fig.13:**
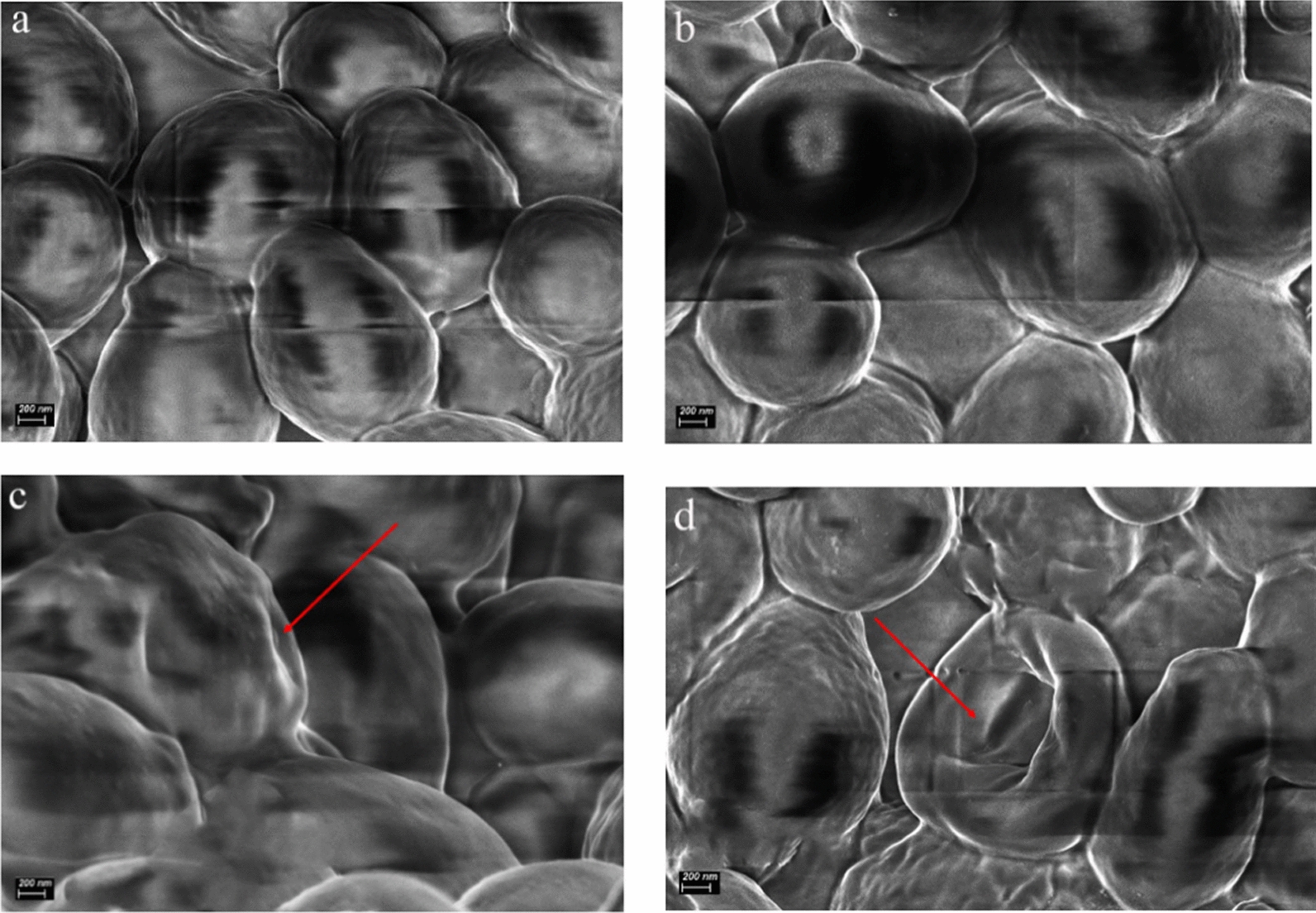
SEM characteristics (**a** *Saccharomyces cerevisiae* without any treatment (control); **b** *Saccharomyces cerevisiae* with thermal treatments (50 $\mathrm{^\circ{\rm C} }$); **c** *Saccharomyces cerevisiae* with PEF treatments (25 $\mathrm{kV}/\mathrm{cm}$); **d** *Saccharomyces cerevisiae* with PEF and thermal treatments (25 $\mathrm{kV}/\mathrm{cm}+$ 50 $\mathrm{^\circ{\rm C} }$))

We took *Saccharomyces cerevisiae* as an example to observe the changes in surface morphology after thermal treatment, PEF treatment, and combined thermal-PEF treatment. The pulse width was 0.5 $\mathrm{\mu s}$, the pulse number was 600, and the frequency was 10 Hz. Before performing SEM, the sample were fixed with 2.5 $\mathrm{\%}$ glutaraldehyde phosphate buffer and kept at 4 $\mathrm{^\circ{\rm C} }$ for 12 h. For Fig. 13a, the sample was smooth, integrated and imporous which was used as a control without any treatments. The second sample was treated by pure thermal treatments at 50 $\mathrm{^\circ{\rm C} }$ for 1 min. The second sample was not obviously different from the first sample on the surface, and it was also smooth, integrated and imporous from Fig. 13b. The third sample treated by pure PEF had a rough, incomplete and porous surface, which formed a sharp contrast with the first two samples from Fig. 13c. The surface of the sample treated with PEF + 50 $\mathrm{^\circ{\rm C} }$ was rougher, more incomplete and more porous from Fig. 13d. It could be seen that the damage degree of cell membrane was greater and the electroporation degree was higher. The surface morphology of the samples treated in different ways was obviously different, which showed that different methods had different degrees of damage to *Saccharomyces cerevisiae*. Increasing the initial temperature, PEF increased the degree of damage to the microbial morphology, and the corresponding microbial survival rate was lower.

## Discussions

This article studied the effects of PEF inactivation on *Saccharomyces cerevisiae*, *Escherichia coli*, and *Bacillus velezensis* at different initial temperatures. Increasing the initial temperature could effectively decrease microbial survival rate by PEF, and avoid dielectric breakdown caused by excessive electric field intensity. At the same time, the article studied the temperature rise of the liquid under the action of different electric field intensities and initial temperatures. The higher the electric field intensity, the greater the temperature rise, which was negative for liquid food. Therefore, it was necessary to weigh in the actual application process to match different initial temperatures and electric field intensities to meet specific requirements. The result of linear fitting proved that temperature could reduce the critical electric field intensity for microorganism inactivation. Therefore, increasing the initial temperature could effectively reduce the survival rate of microorganisms. What is more, the synergy coefficient *k* was bigger than 1, which proved the synergistic effect between temperature and PEF.

The content of nucleic acid and protein in the suspension was negatively correlated with the survival rate of microorganisms. This was because the more inactivated microorganisms, the more substances overflow from the cells. We had verified that temperature and electric field could promote the diffusion of molecules and destroy the steady state of cells through the simulation of molecular transport. This article systematically studied the effects of different initial temperatures (room temperature, 30 $\mathrm{^\circ{\rm C} }$, 40 $\mathrm{^\circ{\rm C} }$, 50 $\mathrm{^\circ{\rm C} }$) and different electrical parameters (electric field intensity, pulse number, and pulse number) on different microorganisms (*Saccharomyces cerevisiae*, *Escherichia coli*, *Bacillus velezensis*). These microorganisms include fungi, bacteria, prokaryotic, eukaryotic, gram-positive bacteria, gram-negative bacteria, bacteria with spore structures, and bacteria without spore structures, proved the universality that temperature could promote inactivation of microorganisms by PEF.

In this paper, we had studied the influence of temperature on microbial inactivation by PEF from the perspective of microbial inactivation rate, critical electric field intensity for microbial inactivation, synergy coefficient, nucleic acid content and protein content in suspension, and molecular transport. We could see that there were many articles explaining the promoting effect of temperature on the inactivation of microorganisms by PEF, including phase change (Liu and Conboy 2004), cell membrane fluidity (Kanduser et al. 2008), cell membrane permeability (Blicher et al. 2009). Through molecular dynamics simulation, it was proved that thermally-driven biological reaction and electrically-driven biological reaction have the same nature. The increase of temperature could supplement the electrically-driven biological effect of membrane and promote electroporation (Song et al. 2011). The formation of nanopores was demonstrated in the presence of a temperature gradients, and it suggested a greater role of temperature gradients in synergistically enhancing electroporation process (Song et al. 2017). Of course, the mechanism of temperature promoting the inactivation of microorganisms by PEF was complicated and unclear, and it needed further research and exploration.

## Data Availability

All data generated or analysed during this study are included in this published article.

## Abbreviation

PEF: Pulsed electric fields
